# A troubled heart: Mood disorder history longitudinally predicts faster cardiopulmonary aging in breast cancer survivorship

**DOI:** 10.1371/journal.pone.0283849

**Published:** 2023-03-31

**Authors:** Annelise A. Madison, Marie Filatov, Rebecca Andridge, Garrie Haas, Stephen P. Povoski, Doreen M. Agnese, Maryam Lustberg, Raquel E. Reinbolt, Robert Wesolowski, Nicole O. Williams, William B. Malarkey, Janice K. Kiecolt-Glaser

**Affiliations:** 1 Institute for Behavioral Medicine Research, The Ohio State University College of Medicine, Columbus, OH, United States of America; 2 Department of Psychology, The Ohio State University, Columbus, OH, United States of America; 3 Division of Biostatistics, The Ohio State University, Columbus, OH, United States of America; 4 Department of Cardiovascular Medicine, The Ohio State University Wexner Medical Center, Columbus, OH, United States of America; 5 The Ohio State University Comprehensive Cancer Center, Columbus, OH, United States of America; 6 Center for Breast Cancer, Yale Cancer Center, New Haven, CT, United States of America; 7 Department of Internal Medicine, The Ohio State University College of Medicine, Columbus, OH, United States of America; 8 Department of Psychiatry and Behavioral Health, The Ohio State University College of Medicine, Columbus, OH, United States of America; Local Health Authority Caserta: Azienda Sanitaria Locale Caserta, ITALY

## Abstract

**Objective:**

Breast cancer survivors live longer due to more advanced cancer treatments; however, cardiovascular disease (CVD) is the leading non-cancer cause of death in breast cancer survivors. Previous studies have shown that depression is associated with an increased risk of CVD development. This study investigated whether depressive symptoms or mood disorder history, either independently or in combination with cardiotoxic treatments, predicted older cardiopulmonary age using a novel index–the Age Based on Exercise Stress Test (ABEST)–among breast cancer survivors.

**Methods:**

Breast cancer survivors (*N =* 80, ages 26–72, stage I-IIIA) were assessed an average of 53 days (*SD* = 26) post-surgery, but before adjuvant treatment, and again an average of 32 (*SD* = 6) months thereafter. At both visits, they reported depressive symptoms on the Center for Epidemiologic Studies Depression Scale (CES-D), completed the Structured Clinical Interview for DSM-V, and engaged in an exercise stress test to obtain ABEST scores.

**Results:**

Controlling for treatment type, age, education, trunk fat, antidepressant use, and time between visits, longitudinal analyses showed that breast cancer survivors with a mood disorder history had worsening ABEST scores over time, compared to their peers without this history (p = .046). Change in physical activity between Visits 1 and 2 did not mediate this relationship (95% CI: -0.16–0.51). Ancillary analyses provided some additional support for the primary finding, such that those with a mood disorder history trended toward greater decreases in Vo_2_max, although results were marginally non-significant *(p =* .095). There were no cross-sectional relationships between depressive symptoms or mood disorder history and ABEST scores (*p*s>.20). Treatment type did not modulate observed relationships (*ps>*.22).

**Conclusions:**

Breast cancer survivors with a mood disorder history may experience faster cardiopulmonary aging compared to their peers without such a history, raising risk for CVD.

## Introduction

Due to more advanced and targeted treatments, breast cancer survivors are living longer. Five-year survival among younger women with breast cancer increased from 74% to 89% between 1975 and 2015 [1]. As survival rates improved, survivors’ health became a major focus, and a concerning pattern emerged regarding the cardiovascular health of breast cancer survivors: Seven years after recovery, cardiovascular morbidity was twice as high among breast cancer survivors as those without a cancer diagnosis [2]. In one study, cardiovascular disease (CVD) accounted for 35% of the mortality in survivors over 50 [2]. Indeed, CVD has become the leading non-cancer cause of mortality among breast cancer survivors [3]. Therefore, identifying breast cancer survivors’ risk factors for cardiovascular morbidity has notable clinical implications.

### The centrality of treatment type

Treatment type is a known risk factor for cardiovascular morbidity and mortality. Chemotherapy, particularly anthracyclines (e.g., doxorubicin), is a standard adjuvant treatment for breast cancer, with about one in three women receiving anthracyclines [4]. Despite their effectiveness in fighting cancer, they have a well-documented cardiotoxic consequences, including both systolic and diastolic dysfunction [5, 6]. Similarly, radiation is a risk factor for CVD [7]. For example, one study of breast cancer survivors found the risk of a major coronary event increased linearly with the mean dose of radiation, with an overall rate of 7.4% increase in major coronary events per unit of absorbed ionizing radiation [8]. Therefore, cancer treatment type is a fundamental consideration when evaluating cardiovascular risk.

### Depression and poorer cardiovascular outcomes

Depression disproportionality affects breast cancer survivors [9]. A meta-analysis of 72 studies across 30 countries found that almost one in three breast cancer survivors suffered from clinically elevated depressive symptoms, compared to only 13% in the general population [10, 11].

Among non-cancer populations, both clinical depression and self-reported, continuously-measured depressive symptoms are risk factors for cardiovascular morbidity and mortality [12–14]. One meta-analysis showed depressed individuals had a 30% increased risk of coronary heart disease and myocardial infarction compared to their nondepressed peers [13]. A more recent study found depressed individuals without coronary heart disease were 1.5 times more likely to be hospitalized for diastolic heart failure over a median of nine years’ follow-up [14]. Additionally, longitudinal evidence among non-cancer populations shows that those with a history of manic and hypomanic episodes were also at a higher risk for cardiovascular disease (odds ratio: 2.97, 95% CI: 1.40–6.34) [15]. Poorer health behaviors, physiological dysregulation (e.g., autonomic imbalance, systemic inflammation), higher incidence of relevant physical comorbidities, or psychotropic medication usage may help to explain why depressive disorders and self-reported depressive symptoms predict poorer cardiovascular outcomes [16].

### The current study

The current study investigated whether self-reported depressive symptoms and mood disorder history were risk factors for faster cardiopulmonary aging, as assessed via a novel index derived from an exercise stress test (EST), detailed below. Additionally, we looked at the effects of cancer treatment type on the relationship between mood disorder history or self-reported depressive symptoms and cardiopulmonary aging. We hypothesized that higher self-reported depressive symptoms or a mood disorder history would be associated with higher ABEST scores cross-sectionally and longitudinally, and that cardiotoxic cancer treatments (i.e., anthracyclines or radiation) would enhance this effect. We also predicted that cardiopulmonary age would increase between post-surgery and post-adjuvant treatment (Visit 1 and Visit 2), due to the combination of cancer- and treatment-related stress, the previously established cardiotoxic effect of some cancer treatments, and the effect of aging.

## Methods

### Participants

**Fig 1:**
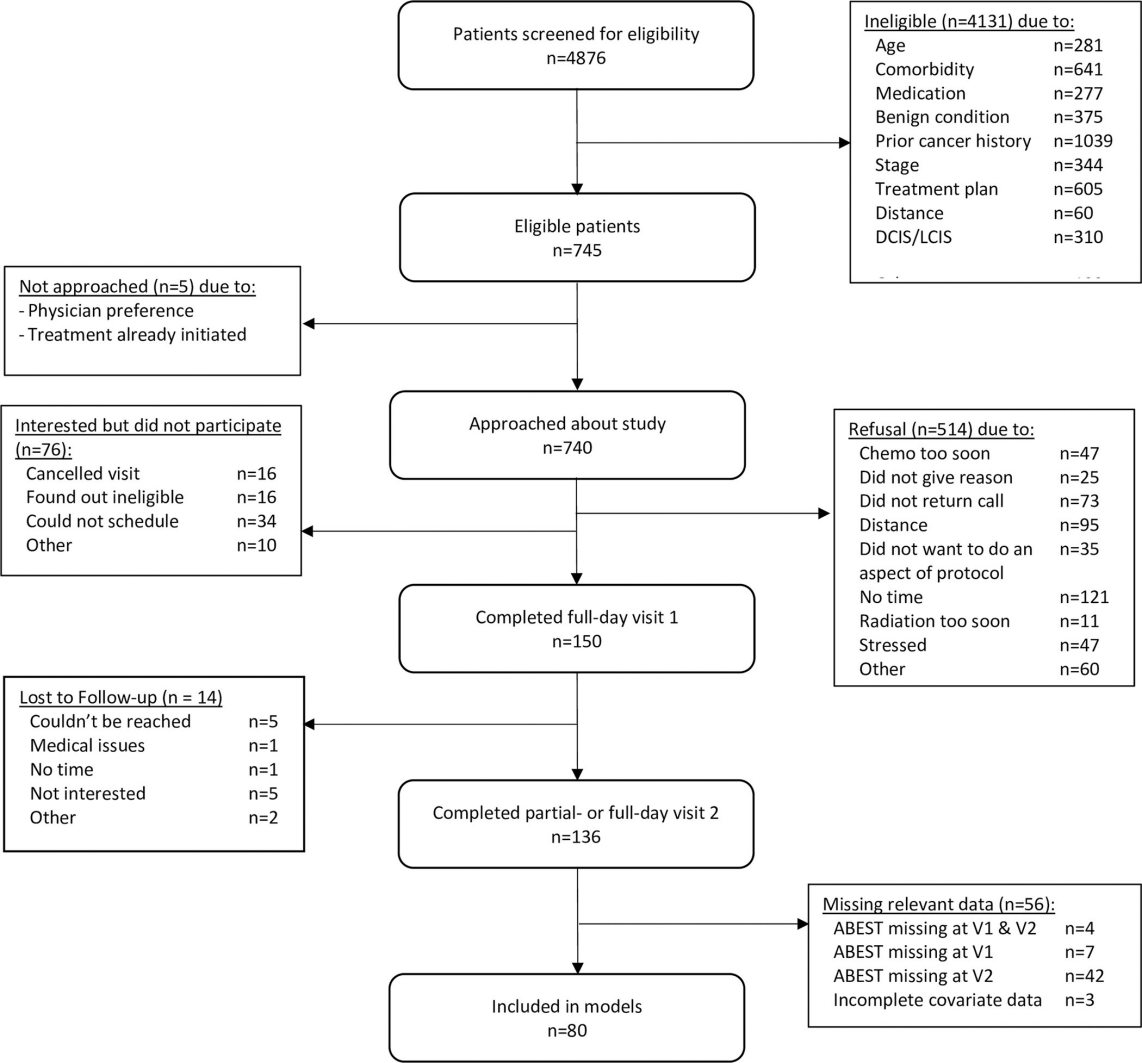
Flowchart of participants through study.

Women (*N =* 80) were diagnosed with stage I-IIIA breast cancer and were recruited post-surgery and pre-adjuvant therapy after staging was known and treatment decisions were made. They were recruited through the Stefanie Spielman Comprehensive Breast Center at The Ohio State University. Exclusions included age (< 21 or > 75), distance from laboratory (> 100 miles), a prior history of any malignancy except basal or squamous cell skin cancer, neoadjuvant chemotherapy or radiation treatment, stroke, diabetes, current heart disease or uncontrolled hypertension, peripheral vascular disease, prior heart attack, heart failure, heart transplant or other major cardiovascular surgery, liver disease, autoimmune and/or inflammatory diseases, alcohol or drug abuse, steroid use, recent (<3 months) initiation of antidepressant medication, and other medical conditions that would limit participation (e.g., dementia). See Fig 1 for the flow of participants throughout the study. Note that although 150 participants completed the full Visit 1 protocol, only 80 had all the data necessary for the current analyses. Overall, 14 women were lost to follow-up, and 56 did not have the requisite data for these analyses. For 12 of these 56 women, the COVID-19 pandemic prevented completion of the second EST due to hygienic concerns related to the EST protocol (i.e., wearing the face mask). Other reasons that some individuals did not complete the second EST include: non-cancer medical or orthopedic issues (*n =* 9), cancer recurrence or metastasis (*n =* 3), life circumstances such as moving or caregiving (*n =* 7), or refusal to complete a second EST (*n =* 15). Also, 10 women were either missing covariate data or EST data from Visit 1. The Ohio State University Institutional Review Board approved the study, and participants provided written informed consent.

### Procedure

Between 2014 and 2021, participants completed two visits–one soon after surgery and the other two to four years later (*M =* 32 months, *SD =* 6, range: 24–51). During each visit, participants reported their depressive symptoms, engaged in a structured psychiatric interview, and completed an EST.

#### Exercise stress test

ESTs can identify subtle changes in cardiopulmonary age even in asymptomatic populations [17]. A trained exercise physiologist conducted a graded cycle ergometer test using an Excalibur cycle ergometer (Lode B.V., Groningen, The Netherlands). This test is appropriate for people of all ages and exercise experience [18]. Prior to the test, participants’ oncologists cleared them for testing. During the test, the exercise physiologist instructed participants to cycle at a constant speed (between 50 and 60 revolutions per minute), and resistance was increased every two minutes. Participants continued exercising until they felt that they could no longer do so or reached an exercise time of 15 minutes. In this subsample, the mean exercise time was 9.26 (*SD =* 2.01) minutes. This test provided an index of cardiopulmonary fitness via participants’ peak oxygen consumption (Vo_2_max). Also, participants completed the EST while wearing a Firstbeat BodyGuard 2 device (Firstbeat Technologies Ltd, Jyväskylä, Finland) and a breathing mask. The heart monitor collected heart rate data throughout the test including peak heart rate and heart rate one-minute post-exercise.

#### ABEST score

The Age Based on Exercise Stress Test (ABEST) incorporates three widely used, objective indicators of EST performance: (1) metabolic equivalent of task (MET), the objective measure of energy expenditure relative to body mass; (2) chronotropic reserve index (CRI), a standard measure of heart rate increment during exercise; and (3) heart rate recovery (HRR), the difference between peak heart rate during exercise and heart rate one minute post-exercise. Low levels of all three are associated with higher mortality risk [19, 20]. Compared to chronological age, ABEST was a better predictor of all-cause mortality among a middle-aged, non-cancer sample [19]. Also, ABEST scores may be a helpful tool to use among patients to promote health behavior engagement, as people understand ABEST scores better than the Framingham risk score and other common metrics [19]. Data obtained from the EST were used to calculate ABEST scores. Individual components were calculated as described in the initial ABEST publication [19]. ABEST scores were derived via the established formula:

ABEST = 63.637–1.861 METs + 4.858 CRI + 3.633 (abnormal HRR) + 1.316 (beta-blocker use) + 2.226 (non-dihydropyridine calcium antagonist use).

#### Depressive symptoms and mood disorder history

The 20-item Center for Epidemiological Studies–Depression (CESD) assessed frequency of depressive symptoms in the past week, ranging from not at all ‘0’ to nearly every day ‘3’ for a maximum score of 60 (Visit 1 Cronbach’s α = .74, Visit 2 α = .85) [21]. Well-trained research personnel administered the Structured Clinical Interview for the DSM -5 (SCID-V) to assess current and lifetime mood disorder history. The diagnostic team reviewed each SCID-V interview in consensus meetings to obtain diagnoses. If a discrepancy between the interview and the rest of the diagnosticians was present following the meeting, the licensed clinical psychologist provided a recommendation. Lifetime history of Major Depressive Disorder, Persistent Depressive Disorder, Bipolar Disorder, Cyclothymia, and Other or Unspecified Depressive Disorder were collapsed to index mood disorder history.

#### Covariates

Covariates were selected a priori following a review of the literature. A whole-body dual x-ray absorptiometry (DXA) scan provided data on trunk fat [22], as central adiposity increases risk for cardiovascular morbidity [23]. Cardiotoxic treatment history was attained via medical chart review (doxorubicin: yes/no, radiation: yes/no) [5, 7]. Other relevant covariates included age [24] and education as a proxy for socioeconomic status (some or all of high school, some college, college graduate, graduate or professional training) [25]. To be sure that any observed relationships were not due to antidepressant medication, we additionally controlled for antidepressant usage, which was obtained from a self-reported medication list at each visit and coded as a single dichotomous variable (yes/no).

#### Physical activity

We measured physical activity due to our interest in whether it mediated effects on cardiopulmonary aging. At each visit, participants reported their physical activity levels on the Godin-Shephard Leisure-Time Physical Activity Questionnaire, which is an easy-to-administer, three-item assessment of participants’ physical activity [26]. The measure is weakly correlated with percentile VO2max (*r =* 0.24, *p <* .001), and percentile body fat (*r =* 0.13, *p <* .01), and can classify individuals as fit or unfit with 69% accuracy [26]. The questionnaire asks participants how often and for how long in the past week they did strenuous, moderate, and mild intensity exercises, with examples provided for each category. Based on physical activity guidelines, Godin proposed an updated scoring system to utilize only the moderate and strenuous (but not mild) components of the Godin because of the known health benefits of exercise that is at least of moderate intensity [27]; accordingly, we computed the met adjusted activity score based on the vigorous and moderate (but not mild) activity.

#### Analytic method

Primary models only include participants with ABEST scores at both visits. After completing the EST at Visit 1, 42 participants opted to complete a partial Visit 2 protocol, which did not include the EST. Fourteen others were lost to follow-up, and an additional 14 were missing other relevant data. Therefore, only 80 women were included in our models. We performed unequal variance t-tests and Fisher’s exact tests to compare those who were included versus excluded from models. Those who were excluded from the analysis sample were more likely to have a mood disorder history at Visit 1 (44% vs 26%, *p* = .03) and had higher Visit 1 ABEST scores (*t*(122.3) = 3.31, *p* = .001).

In line with our hypotheses, we first used paired t-tests to test whether ABEST scores and its constituent components changed over time. We then used a linear mixed effects model with an unstructured covariance matrix for the repeated visits and Kenward-Rogers adjustment to the degrees of freedom to model ABEST scores at both visits and used as predictors 1) continuous depressive symptoms and 2) mood disorder history, in separate models. Next, we used linear regression models controlling for Visit 1 ABEST scores to predict change in ABEST scores from Visit 1 to Visit 2 using depressive symptoms at Visit 1 and mood disorder history at Visit 1 or anytime between Visit 1 and Visit 2 as the primary predictors of interest in separate models. Lastly, we added two interaction terms between the predictor of interest and cardiotoxic treatment type (doxorubicin or radiation treatment, as separate interaction terms) to examine whether the effect of depression on changes in ABEST scores depended on treatment type. Primary models controlled for age (time-varying), doxorubicin treatment, radiation treatment, education, trunk fat (time-varying), antidepressant use, and time between visits (longitudinal models) or time since surgery (cross-sectional models).

Of note, only one of our participants had a bipolar disorder history, and none had a history of cyclothymia, so we conducted sensitivity analyses with this participant excluded, but our pattern of results remained unchanged. As a post-hoc test, we used the PROCESS macro to test whether change in physical activity mediated significant relationships. PROCESS uses bootstrapping to estimate the indirect effect as a test of mediation, and we used 5,000 bootstraps to generate an indirect effect and confidence interval [28]. For all analyses, alpha levels were set at .05. All analyses were conducted in SAS version 9.4 (Cary, NC). Data are available from the corresponding author on reasonable request.

## Results

### Demographic information

Women included in the analysis sample ranged in age from 26 to 72 (*M =* 51.36, *SD =* 9.79) and tended to be highly educated with 61% earning at least an undergraduate degree. The sample was mostly White (84%) and non-Hispanic (95%). Of the four individuals who identified as Hispanic, three identified as White and one identified as mixed race. In terms of treatment, 39% had chemotherapy, 13% had doxorubicin, 60% had radiation, 46% had a lumpectomy, and 54% had a mastectomy. Doxorubicin was the only anthracycline used among our sample. The surgeries occurred an average of 53 days (SD = 26) prior to Visit 1 (range: 22 to 139). In terms of staging, 48% of women had Stage 1 cancer.

Just over a quarter of our sample had a mood disorder history, 19% were taking antidepressant medication, and the average CESD score was 8.70 (SD = 5.51, range: 0–29) at Visit 1. The same number of individuals took antidepressant medication at Visits 1 and 2 (*n =* 23), but only 12 reported taking antidepressant medication at both visits, three discontinued the medication between visits, 11 began taking medication after Visit 1, and 54 did not report antidepressant usage at either visit. Of the 21 who had a mood disorder history at Visit 1, 18 had major depressive disorder (with one also meeting criteria for persistent depressive disorder), one had bipolar disorder, one had persistent depressive disorder and had a major depressive episode between Visits 1 and 2, and one had other specified depression and also had a major depressive episode between Visits 1 and 2. In total, nine of these individuals had a recurrent episode between Visits 1 and 2. Five additional individuals who did not have a mood disorder history at Visit 1 had a major depressive episode between Visits 1 and 2.

The average ABEST score (*M =* 57.17, *SD =* 3.23, range: 45.11–64.27) was higher than the average age. Also, 19% of women had abnormal heart rate recovery, defined as fewer than 12 beats per minute. A paired t-test revealed that across the sample, there was no change in physical activity from Visit 1 (Visit 1: *M =* 19.84, *SD* = 19.23) to Visit 2 (Visit 2: *M =* 21.29, *SD =* 20.79, *p =*, 53). See Table 1 for further demographic information. Table 2 provides the zero-order correlation matrix for the Visit 1 variables of interest.

**Table 1:** Demographics of analysis sample at Visit 1.

Variable		N	%	Mean (SD)	Range	
Abnormal HRR	Yes	15	19%			
	No	65	81%			
ABEST				57.17(3.23)	45.11–64.27	
CRI				0.91(0.14)	0.53–1.26	
HRR				22.28(10.92)	4–68	
METS				6.28(1.64)	3.31–12.34	
Age				51.36(9.79)	26.08–72.17	
Godin Activity Score				19.84(19.23)	0–79	
Mood Disorder History	None	59	74%			
	Past	20	25%			
	Current	1	1%			
CESD				8.70(5.51)	0–29	
Trunk Fat (kg)				15.50(6.58)	4.19–30.36	
Antidepressant use	Yes	15	19%			
	No	65	81%			
Education	High School	11	14%			
	Some College	20	25%			
	College Degree	24	30%			
	Grad/Prof Training	25	31%			
Race	White	67	84%			
	Black	7	9%			
	Asian	1	4%			
	Native American	1	1%			
	Mixed Race	2	2%			
Ethnicity	Not Hispanic	76	95%			
	Hispanic	4	5%			
Doxorubicin treatment	Yes	10	13%			
	No	70	88%			
Radiation treatment	Yes	48	60%			
	No	32	40%			
Surgery Type	Mastectomy	43	54%			
	Lumpectomy	37	46%			
Days Since Surgery				53.03(26.47)		22–139
Cancer Stage	1	38	48%			
	2	41	51%			
	3	1	1%			

CESD = Center for Epidemiological Studies Depression Scale; CRI = chronotropic reserve index; MET = metabolic equivalent of task; HRR = heart rate recovery; ABEST = Age Based on Exercise Stress Test

**Table 2:** Zero-order Pearson correlations among variables of interest at Visit 1.

	2	3	4	5	6	7	8	9	10	11	12	13	14	15
1. Age	.15	-.08	-.09	-.03	-.20	-.02	.12	-.12	.02	-.14	.15	**-.39**	.09	**.36**
2. Trunk Fat		-.01	.08	**-.32**	.00	**.32**	.15	.12	**-.40**	.09	-.17	**-.70**	-.05	**.59**
3. CESD			**.23**	-.06	.02	-.09	-.13	-.03	-.11	-.09	.08	.01	-.05	.00
4. Mood Disorder History				-.10	-.14	.08	**-.23**	.15	.05	.05	.03	-.01	.22	-.08
5. Education					-.14	**-.24**	.00	.01	.09	.05	.11	**.26**	.06	-.15
6. Doxorubicin						.08	**.33**	.11	-.15	-.09	-.12	.03	-.09	-.02
7. Radiation Treatment							-.04	-.07	**-.26**	**-.28**	**-.23**	-.15	-.05	.02
8. Cancer Stage								.06	-.10	.09	.06	**-.23**	.01	.20
9. Antidepressant Usage									-.06	.09	-.01	.00	.09	.04
10. Godin Activity Score										.05	**.16**	**.45**	**.38**	**-.42**
11. Days Since Surgery											.03	-.08	.15	.10
12. CRI												.19	-.05	.07
13. METs													.05	**-.87**
14. HR Recovery														**-.29**
15. ABEST														

CESD = Center for Epidemiological Studies Depression Scale; CRI = chronotropic reserve index; MET = metabolic equivalent of task; HR = heart rate; ABEST = Age Based on Exercise Stress Test; Bold indicates p < .05

### Primary analyses

#### Cross-sectional results

In terms of the ABEST’s individual components, MET (*p =* .75), CRI (*p =* .32), and HRR (*p* = .11) did not change from Visit 1 to Visit 2. ABEST scores were not significantly different at Visit 2 (mean (SD) = 56.84 (3.65)) compared to Visit 1 (mean (SD) = 57.17 (3.23), *p =* .25). Cross-sectionally, depressive symptoms (*p =* .68) and mood disorder history (*p =* .75) were unrelated to ABEST scores. In terms of covariates, older women (*B =* 0.10, *SE =* 0.028, *F(*1, 73.3) = 13.62, *p =* .0004), those with more trunk fat (*B =* 0.31, *SE =* 0.043, *F(*1, 101) = 52.86, *p <* .0001), and those with a shorter time since surgery (*B* = -0.31, *SE* = 0.10, *F*(1,95) = 8.61, *p* = .004) had higher physical ages based on exercise testing, but none of the other covariates predicted ABEST scores cross-sectionally (*p*s>.06).

#### Longitudinal results

**Fig 2:**
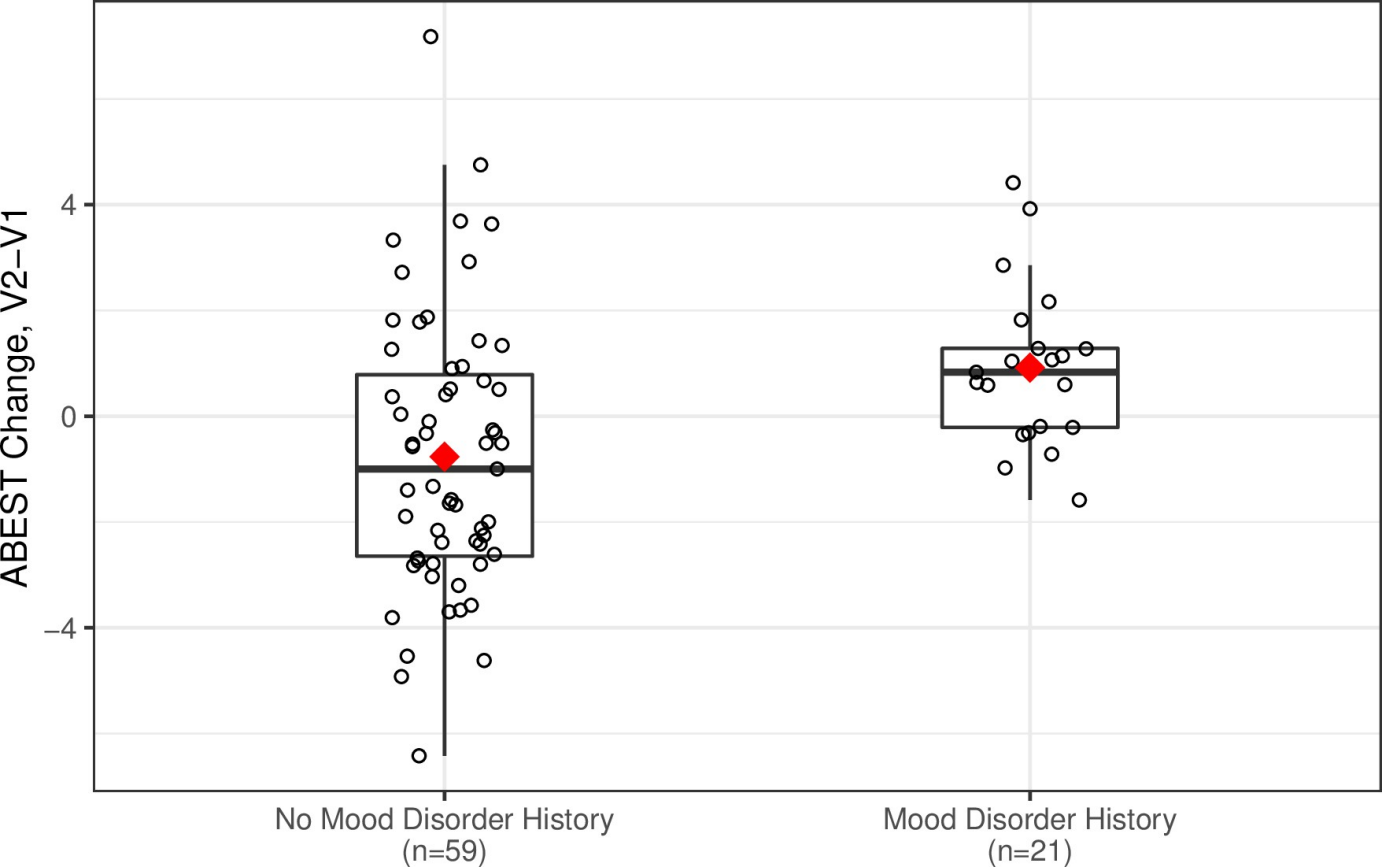
Mood disorder history predicts accelerated cardiopulmonary aging from pre-surgery to post-adjuvant treatment in breast cancer survivors. This boxplot depicts the unadjusted ABEST change scores from Visit 1 to Visit 2 for those with and without a mood disorder. In fully adjusted models, those with a mood disorder history at Visit 1 had greater increases in ABEST scores from pre-surgery to post-adjuvant treatment than those without a mood disorder history (*p =* .046).

On average, women who had a mood disorder history had an increase in ABEST scores from Visit 1 to Visit 2 (n = 26, mean change (SD) = 0.58 (1.69), 95% CI = -.10 to 1.26) while women who did not have a mood disorder history had a decrease in ABEST scores (n = 54, mean (SD) = -0.76 (2.67), 95% CI = -1.49 to -.03). This difference was significant even after controlling for Visit 1 ABEST scores and other relevant covariates (*B =* 1.18, *SE =* 0.58, *t(*67) = 2.03, *p =* .046), see Fig 2. Visit 1 depressive symptoms did not predict change in ABEST scores (*p =* .80). Neither treatment with doxorubicin or radiation modulated the relationships between Visit 1 mood disorder diagnosis or self-reported depressive symptoms and change in ABEST scores over time (*p*s>.42). In terms of covariates, in models without depressive symptoms or mood disorder history, those with lower Visit 1 ABEST scores (*B =* -0.38, *SE =* 0.11, *t(*68) = -3.48, *p* = .0009) and older individuals (*B* = 0.065, *SE* = 0.030, *t*(68) = 2.15, p = 0.04) had greater change in ABEST scores across time. None of the other covariates predicted ABEST change (*ps*>.08), though there was a trend for individuals more trunk fat to have greater change in ABEST scores (*B* = 0.098, *SE* = 0.057, *t*(68) *=* 1.72, p = 0.09).

### Ancillary analysis

To corroborate our primary significant finding–the longitudinal relationship between mood disorder history and change in cardiopulmonary aging–we used the same modeling strategy to test whether mood disorder history predicted Vo_2_max change. On average, women who had a mood disorder history had a decrease in Vo_2_max from Visit 1 to Visit 2 (mean change (SD) = -1.02 (2.73), 95% CI = -2.12 to .08) while women who did not have a mood disorder history did not experience this decline (mean (SD) = .30 (3.93), 95% CI = -.78 to 1.37). This difference was marginally nonsignificant after controlling for relevant covariates (*B* = -1.57, *SE* = .93, *t(*67*) =* -1.69, *p* = .095). Treatment type did not moderate this relationship (*ps*>0.22).

### Post-hoc mediation analysis

Change in physical activity, as measured by the Godin, did not mediate the relationship between mood disorder history and cardiopulmonary aging (95% bootstrapped CI: -0.16–0.51).

## Discussion

Among an age-diverse sample of breast cancer survivors evaluated roughly two months post-surgery as well as over two years post-adjuvant treatment, we used a novel index of cardiopulmonary aging—the ABEST [19] and found that mood disorder history predicted ABEST trajectories from pre- to post-adjuvant treatment. Whereas women without a mood disorder history recovered post-adjuvant treatment (i.e., significant declines in ABEST scores), women with a mood disorder history experienced faster cardiopulmonary aging. Ancillary analyses corroborated our primary finding, demonstrating that those with a mood disorder history also exhibited a trend toward greater declines in Vo_2_max over time. The primary finding is particularly notable when considered in light of our non-significant predictors of cardiopulmonary aging trajectories, including established cardiotoxic cancer treatments (i.e., radiation, anthracyclines). Additionally, the relationship between mood disorder history and cardiopulmonary age only emerged longitudinally–not cross-sectionally–suggesting that a mood disorder history may become physiologically relevant over time across particularly stressful and taxing life events. Also, only mood disorder history, rather than current self-reported depressive symptoms, predicted ABEST trajectories, which shows that symptoms must be of adequate severity and duration to predict cardiopulmonary aging trajectories in cancer survivorship.

### Mood disorder history and cardiopulmonary aging

A mood disorder history may impact post-adjuvant cardiopulmonary aging through physiological and behavioral pathways. For example, clinical depression as well as mildly elevated depressive symptoms can enhance inflammatory responses to stress [29–31], which, over time, may lead to the heightened basal inflammation that is observed in a significant subset of depressed patients [32]. Heightened levels of inflammation, in turn, correspond with blood vessel damage and plaque buildup, ultimately increasing risk for CVD [33, 34]. In terms of behavioral mediators, depressed people are significantly less likely to adhere to lifestyle recommendations, such as physical activity guidelines, and half as likely to follow proper medication management [35]. The problematic health behaviors common in depression are associated with an increased risk for cardiovascular disease, stroke, and heart failure [36]. Even so, in the current study, declines in physical activity did not mediate the relationship between mood disorder history and cardiopulmonary aging, suggesting a need to explore other mechanisms that may connect a mood disorder history with faster cardiopulmonary aging.

### Possible support for the scarring hypothesis

Our findings provide some support for the scarring hypothesis, which asserts that a mood disorder’s physiological and psychological effects may linger even after remission, increasing vulnerability for future depressive episodes. To date, empirical evidence is mixed [37–41]. Of note, scars can wax and wane throughout the lifespan, and failing to account for this dynamicity may lead to mixed and inconclusive results [42]. Specifically, scars may reemerge during the stress of breast cancer, increasing the psychological and physiological toll of breast cancer. For example, those with a mood disorder history may have heightened physiological responses to stress, which, over time, may promote the poorer physiological aging trajectories observed in this study. In essence, mood disorders may leave physiological scars that emerge during disease onset or treatment or other particularly stressful times.

### EST tolerance

As mentioned above, we had a much smaller sample size for the longitudinal analyses than initially expected. There were many reasons for this attrition, such as cancer recurrence or metastasis, orthopedic issues, or moving. Another issue we repeatedly encountered was that ten percent of our participants refused to complete their second EST because they found the first to be aversive. This percentage is likely even higher because we lost 14 additional participants to follow-up, and it is possible that some of these women discontinued participation because they did not want to complete the second EST. This unexpected phenomenon is notable in and of itself, and it is a critical consideration for future research among breast cancer survivors. Generally, breast cancer survivors have poorer cardiovascular fitness than their age-matched peers [43], which can make exercise more aversive, and survivors remain physically inactive after cancer treatment [44]. Indeed, in our sample, the average Godin activity score at each visit was below the cut score of 24 that corresponds with American College of Sports Medicine physical activity guidelines. Even so, one small study found that survivors may tolerate treadmill ESTs better than cycling ESTs [45]. Future longitudinal studies conducting repeated ESTs among breast cancer survivors should factor in the possibility of a substantial refusal rate for repeated testing.

### Strengths and limitations

This novel study examined depression and cardiopulmonary age before and after adjuvant cancer treatment–a potentially sensitive time for breast cancer survivors. A major strength of the study is the unique timing of measurements, and, in particular, the pre-adjuvant timepoint when breast cancer survivors are recovering from surgery and anticipating what is to come. The study included both self-reported depressive symptoms, which captured the dimensional nature of depression, as well as the SCID-V to assess lifetime mood disorder history–other strengths. Additionally, this is the first study to use the ABEST in a cancer population, allowing us to probe changes in cardiopulmonary age even among patients who are otherwise relatively healthy. Of note, given our strict exclusionary criteria, this breast cancer sample primarily had low-grade breast cancer, did not have CVD, and did not have the higher prevalence of current mood disorders often seen in cancer populations. Also, many of our participants, particularly those who were more depressed and had poorer ABEST scores, did not complete the second EST and therefore were excluded from analyses. Our observations may be even more pronounced in a sicker, more depressed breast cancer sample. Along the same lines, this sample was comprised of mostly White, well-educated women, which is not representative of the general population–a limitation; results should be replicated among a more diverse sample. Lastly, one nuance of our sample that does not align with the larger literature was that those with more advanced cancer were less likely to have a mood disorder history than those with earlier stages of cancer (*r = -*0.23). That said, all but one of our participants had Stage I or II cancer, so due to this restricted range, it is problematic to assign too much weight to this finding. Nonetheless, it is notable that those with a mood disorder history had faster cardiopulmonary aging despite having less advanced cancer, indicating that the observed result is unconfounded by more advanced disease.

### Clinical implications

Assessing mood disorder history prior to initiating adjuvant treatment can help to identify those who are at risk for faster cardiopulmonary aging following treatment. Current clinical guidelines recommend depression screening at diagnosis, treatment initiation, and at regular intervals during and after treatment [46]. The current findings underscore the importance of doing so prior to adjuvant treatment initiation as an important predictor of post-treatment cardiopulmonary aging.
